# Adoptive Transfer of Regulatory T Cells as a Promising Immunotherapy for the Treatment of Multiple Sclerosis

**DOI:** 10.3389/fnins.2019.01107

**Published:** 2019-10-15

**Authors:** Samuel S. Duffy, Brooke A. Keating, Gila Moalem-Taylor

**Affiliations:** School of Medical Sciences, University of New South Wales, UNSW Sydney, Kensington, NSW, Australia

**Keywords:** regulatory T cells, multiple sclerosis, experimental autoimmune encephalomyelitis, immunotherapy, adoptive cell transfer

## Introduction

Multiple sclerosis (MS) is a debilitating, chronic inflammatory disorder of the central nervous system (CNS) that is characterized by heterogeneous patterns of neurological symptoms (Compston and Coles, 2002), and is the leading cause of disability in young and middle-aged people in the developed world (Koch-Henriksen and Sorensen, 2010). MS patients exhibit impaired immunoregulatory mechanisms that lead to pathological immune responses and neuroinflammation, however it is unclear whether this dysregulation is the cause or a consequence of the disease. As such, MS treatment most often involves disease-modifying immunomodulatory therapies (IMTs), as well as symptomatic management. IMTs have arguably been the most significant advances in the treatment of MS. There are currently numerous approved IMTs that are effective in reducing the frequency of relapses and slowing the progression of relapsing-remitting MS (Garg and Smith, 2015; Faissner and Gold, 2018) through the suppression of aberrant immune responses facilitated by autoreactive lymphocytes. However, despite the growing therapeutic armamentarium for MS over the past two decades, currently approved therapies are ineffective in a subset of patients with aggressive MS and in progressive forms of the disease.

Adoptive cell transfer therapies have revolutionized the treatment of certain cancers (Yang and Rosenberg, 2016), and this success is now being translated to the treatment of other conditions. The concept of cellular adoptive immunotherapy has recently emerged as an exciting therapeutic approach to treating a variety of diseases with an autoimmune component, including MS (Rosenblum et al., 2015). Here, we outline recent progress relating to adoptive transfer of regulatory T (Treg) cells in the treatment of human autoimmune disease, and discuss the prospects of Treg adoptive cell transfer as a novel treatment in MS and associated symptoms.

## Treg Cell-Based Therapies as a Viable Therapeutic Option

**Figure 1:**
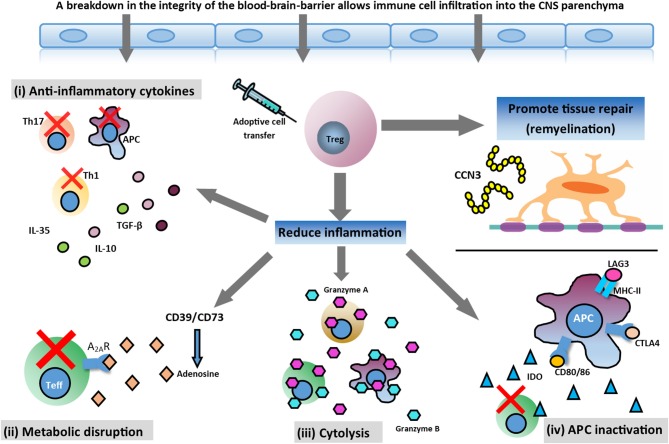
Potential therapeutic action of Treg cell therapy. In the inflamed CNS, Treg cells can mediate immunosuppression and reduce inflammation through various mechanisms and help tissue repair by promoting myelin regeneration. Mechanisms involved in reducing neuroinflammation are: (i) secretion of anti-inflammatory cytokines produced by Treg cells (TGF-β, IL-10, and IL-35); (ii) metabolic disruption ameliorating effector T (Teff) cell function; (iii) cytolysis of Teff cells; and (iv) antigen presenting cell (APC) inactivation. Additionally, Treg cells may promote oligodendrocyte differentiation and remyelination in the damaged CNS through the production of CCN3, a growth regulatory protein.

Treg cells are potently immunosuppressive, and play a pivotal role in regulating the immune system by maintaining self-tolerance and inhibiting autoimmunity (Vignali et al., 2008). Treg cells effectively control the activation, proliferation, and effector functions of key immune cells central to the pathogenesis of MS, such as effector T (Teff) cells, B cells, and antigen-presenting cells (APCs) (Sakaguchi et al., 2008). This is accomplished through several mechanisms, including (i) secretion of inhibitory cytokines, such as interleukin (IL)-10; (ii) metabolic disruption–Treg cells express CD39 and CD73 which catalyse the generation of pericellular adenosine and downregulate Teff cell responses via activation of the adenosine 2A receptor (A2AR); (iii) direct cytolysis through granzyme-mediated killing; and (iv) APC inactivation–Treg cells can modulate APC function by lymphocyte activation gene 3 (LAG-3)/major histocompatibility complex class II (MHC-II)-mediated suppression of cell maturation and cytotoxic T lymphocyte antigen 4 (CTLA-4)/CD80/86-mediated induction of indoleamine 2,3-deoxigenase (IDO), which is an immunosuppressive enzyme (Vignali et al., 2008; Gravano and Vignali, 2012; Bluestone and Tang, 2018; Shevach, 2018). In addition, Treg cells have recently been implicated in potentiating tissue repair. In the CNS, Treg cells have been shown to directly drive remyelination, independent of immunomodulation, through the elaboration of the growth regulator CCN3 (Dombrowski et al., 2017; Figure 1).

Myelin self-reactive T cells are present in both MS patients and the healthy population, suggesting that their presence alone is not sufficient for disease induction but requires an aspect of immune dysregulation. This may arise via dysfunctional systemic or local immunoregulatory systems, through either genetic pre-disposition or transiently as a product of concomitant infection (Christoffersson and von Herrath, 2019). Indeed, Treg cell numbers have been reported to be altered in the blood of MS patients, and may possess impaired functional capacity (Duffy et al., 2018). A failure of endogenous Treg cell-dependent immunoregulatory mechanisms thus provides a plausible contributing factor in the initiation and maintenance of neuroinflammation in MS.

Treg cells display unique properties which underscore their potential as viable candidates for cellular therapy in MS. Unlike many conventional therapeutics used to treat MS, Treg cells can effectively traffic to target tissues by following chemoattractant molecules released at the site of inflammation. Treg compartmentalization and trafficking appears to be tissue-specific, with distinct chemokine receptor and integrin expression contributing to selective retention and trafficking of Treg cells at sites where regulation is required (Wei et al., 2006). However, the specific migratory pathways and chemokine receptors involved in the trafficking of Treg cells to the CNS remains to be elucidated. Treg cells also have an extended half-life *in vivo* and can self-regulate their number and function depending on therapeutic demand in the target tissue (Bluestone and Tang, 2018). Treg cells have no requirement to directly contact autoreactive immune cells to exert their suppressive effects, as they are able to alter the local inflammatory milieu through the expression of cell surface receptors and the elaboration of soluble mediators such as the anti-inflammatory cytokines transforming growth factor (TGF)-β, IL-10, and IL-35 (Duffy et al., 2018). These cytokines may also facilitate the emergence of additional immunosuppressive cell subtypes in a process termed “infectious tolerance” (Gravano and Vignali, 2012). In a recent study utilizing the experimental autoimmune encephalomyelitis (EAE) animal model of MS, we showed that intrathecal administration of IL-35 alleviated disease progression and pain behaviors in mice, and induced the development of a subpopulation of T and B effector cells that produced the anti-inflammatory molecule IL-10 (Duffy et al., 2019). Such generation of additional immunosuppressive cell populations may serve to amplify and prolong the effects of Treg cell-based therapies, even if the originally introduced Treg cells fail to survive indefinitely *in vivo*. The neurodegenerative component of MS is challenging to treat and effective therapies for progressive forms of the disease are lacking. Interestingly, Treg cells have been shown in animal models to promote CNS remyelination (Dombrowski et al., 2017), which suggests an additional neuroprotective role for the Treg cell in addition to their known immunomodulatory actions. In support of this, we have recently demonstrated that spinal delivery of both adoptively-transferred Treg cells and IL-35 decreased CNS demyelination in mice with EAE, and this was associated with reduced pain behaviors (Duffy et al., 2019).

Treg cell adoptive transfer has many advantages over currently-available MS drug therapies including: (i) autologous transfer of patients' own cells which have potential to self-regulate depending on therapeutic demand, (ii) durability of immunosuppression compared to many conventional drug therapies, (iii) exciting potential to treat neurodegeneration and key symptoms (e.g., chronic pain) in MS, and (iv) the possibility of targeted and highly personalized therapies utilizing *ex vivo* modification of cells according to specific patient profiles. Building on the promising outcomes of pre-clinical animal research, several clinical studies have demonstrated safety and tolerability of Treg cell adoptive transfer in conditions including type 1 diabetes (Bluestone et al., 2015), graft vs. host disease (Brunstein et al., 2016), and amyotrophic lateral sclerosis (ALS) (Thonhoff et al., 2018), and further clinical trials are currently underway. Efficacy has been suggested in ALS, where autologous Treg cells that were expanded *in vitro* and administered intravenously with concomitant IL-2 slowed progression rates during early and later stages of disease (Thonhoff et al., 2018).

## Limitations of Treg Cell-Based Therapies

Although adoptive transfer of Treg cells is an attractive approach for the treatment of MS, there are several potential limitations for Treg cell-based therapies:

### Dysfunctional Patient Treg Cells

Recent studies have suggested impaired functioning of Treg cells derived from MS patients, as well as an unresponsiveness of Teff cells to Treg cell-mediated suppression (Schneider et al., 2013; Duffy et al., 2018). This holds implications for autologous transfer of Treg cells, as any deficit in the functional capacity of Treg cells derived from MS patients will likely need to be corrected before reinfusion if the therapy is to be effective. Interestingly, ALS patients have also been shown to display dysregulation of Treg cells, which was reversed following *ex vivo* expansion with rapamycin/IL-2 and intravenous Treg cell adoptive transfer with concomitant subcutaneous IL-2 injection. However, the mechanisms underlying this change in phenotype are unknown (Beers et al., 2017; Thonhoff et al., 2018). An alternative approach would be to utilize gene editing to delete polymorphic human leukocyte antigens on fully functional Treg cells derived from unrelated patients. This approach is currently being utilized using adoptive transfer of Teff cells in some cancers (Bluestone and Tang, 2018).

### Required Dose and Subset of Treg Cells

What constitutes a therapeutic dose of treg cells remains unclear. The therapeutic dose in a given disease setting likely depends on Treg potency, disease state and activity, and whether protocols employ polyclonal or antigen-specific Treg cells. It is also important to avoid undesirable levels of systemic immunosuppression, which may pre-dispose to infection and malignancy (Singer et al., 2014). Moreover, there are several distinctive subtypes of Treg cells, including thymus-derived/natural Treg (nTreg), inducible/adaptive Treg (iTreg), IL-10-producing type 1 regulatory (Tr1), and CD8+ treg cells (Shevach and Thornton, 2014; Roncarolo et al., 2018) and it is unknown which precise subset should be selected and expanded in order to achieve the most desirable outcome. Selection of human nTreg cells based on the phenotypic expression of CD4, CD25, and the transcription factor FoxP3, can be problematic since it may, but not always, correlate with their immunoregulatory capacity, and FoxP3 can be transiently expressed in Teff cells (Walker et al., 2003). Other markers, including CD25+ and CD127−, are currently being used to circumvent this problem, however it is unknown whether this population encompasses the most disease-relevant subset for therapy (Christoffersson and von Herrath, 2019). Further research is needed to outline the roles of these subsets in MS, and the most optimal treg cell subset for selection and expansion.

### Antigen-Specificity

The specific antigenic target in MS is unclear, and an epitope-spreading process has been implicated in MS (Riedhammer and Weissert, 2015). MS patients often display autoantibodies to multiple myelin and neuronal components which may vary between patients (Somers et al., 2008). Identification of causative epitopes in an individualized setting is problematic, which makes induction of antigen-specific tolerance in MS challenging. adoptive transfer of polyclonal Treg cells with broad antigen specificity may be less technically challenging and allow for administration of a greater number of Treg cells, but problems may arise due to off-target tissue effects. Antigen-specific treg cells may provide more localized suppression of harmful immune responses in the CNS and/or draining lymph nodes in MS. As such, further research is needed to identify candidate driver antigens in MS to achieve the maximum effect of Treg cell-based therapies. Chimeric antigen receptor T cell (CAR-T) technology is an approach with origins in the oncology field, and has been used to generate Treg cells expressing T cell receptors specific for antigens relevant to certain pathologies for transfer into recipients (Zhang et al., 2018). Limited research exists on the use of CAR-based technologies in EAE. However, engineered Treg cells overexpressing a CAR targeting myelin oligodendrocyte glycoprotein *in trans* with the murine FoxP3 gene demonstrated suppressive capacity *in vitro*. Following intranasal delivery, these cells effectively trafficked to various regions of the brain, and suppressed clinical EAE and neuroinflammatory changes (Fransson et al., 2012). Further, CAR-T technology has been used to program human Treg cells to express transgenic T cell receptors specific for myelin basic protein, which were also immunosuppressive and disease-ameliorating in EAE (Kim et al., 2018). This provides promising evidence that human Treg cells may be programmed to possess antigen specificity in an autologous setting in the future.

### Treg Cell Instability and Plasticity

Whether treg cells retain their function following transfer *in vivo* remains unknown. Several studies have suggested that Treg cells may become unstable under certain inflammatory conditions, derailing their immunoregulatory role (Delgoffe et al., 2013; Sakaguchi et al., 2013). Considerable plasticity exists in the Treg cell lineage, whereby there is potential for acquisition of alternative effector or hybrid fates, and promotion rather than inhibition of inflammation (Sawant and Vignali, 2014). Bluestone and Tang (2018) theorize that the risks associated with treg cell instability may be mitigated by programming adoptively-transferred Treg cells to encode suicide genes, to secrete autocrine IL-2, deleting receptors for proinflammatory cytokines, or by stabilizing FoxP3 expression (Bluestone and Tang, 2018).

### Potential Harmful Side Effects

At high doses, Treg cells may cause severe immunosuppression and a compromised immune system, which may lead to increased vulnerability to life-threatening opportunistic infections and increased risk of developing malignancies. Another potential hurdle relates to the apparent dichotomy of Treg cell function in certain disease contexts within the CNS. Although treg cells inhibit neuroinflammation, they also might suppress protective Teff cell responses that may act to limit neurodegeneration (Duffy et al., 2018). MS is a highly heterogenous condition with numerous subtypes each displaying unique clinical courses and underlying pathological processes, which has important implications for a Treg cell-based treatment. As such, further research is necessary to determine the potential efficacy of treg cell adoptive transfer across MS subtypes and different stages of disease progression.

## Conclusions and Future Directions

Treg cells have the ability to mediate immunosuppression and promote regeneration by acting in both lymphoid organs and the inflamed CNS in MS (Figure 1), making them viable candidates for adoptive cell therapy. Further research into Treg cell function, numbers, tolerated dose, antigen-specificity, stability, and timing of adoptive transfer strategies will undoubtedly improve the design of Treg cell-specific therapeutic options for patients with MS. It is likely that a combination approach of Treg cell-based therapies alongside conventional drugs will prove superior to monotherapy in MS (Baecher-Allan et al., 2018). The efficacy of such approaches will be highly dependent on the pathological subtype and clinical phenotype of the disease, meaning a personalized approach to treatment will be necessary. Nevertheless, Treg cell adoptive transfer provides an exciting prospect for the future treatment of MS and its comorbidities.

## Author Contributions

SD conceived the idea and wrote the manuscript. BK prepared the figure and wrote the manuscript. GM-T conceived the idea, designed the figure, and revised and edited the manuscript. All authors have approved the paper.

### Conflict of Interest

The authors declare that the research was conducted in the absence of any commercial or financial relationships that could be construed as a potential conflict of interest.
